# Δ-Peritoneal Cancer Index (Δ-PCI) to Predict Complete Cytoreduction and Histopathological Response to Neoadjuvant Chemotherapy in Ovarian Cancer

**DOI:** 10.3390/jcm13226915

**Published:** 2024-11-17

**Authors:** Giulia Spagnol, Sofia Bigardi, Michela Zorzi, Matteo Morotti, Massimo Carollo, Giulia Micol Bruni, Orazio De Tommasi, Matteo Tamagnini, Livia Xhindoli, Marco Noventa, Roberto Tozzi, Carlo Saccardi, Matteo Marchetti

**Affiliations:** 1 Unit of Gynecology and Obstetrics, Department of Women and Children’s Health, University of Padua, 35122 Padua, PD, Italy; 2 Department of Oncology, Lausanne University Hospital (CHUV), University of Lausanne (UNIL), 1005 Lausanne, Switzerland; 3 Department of Diagnostics and Public Health, University of Verona, 37129 Verona, VR, Italy

**Keywords:** ovarian cancer, neoadjuvant chemotherapy, peritoneal cancer index, chemotherapy response score, interval cytoreductive surgery

## Abstract

**Objectives:** To analyze the role of PCI variation (Δ-PCI) before and after neoadjuvant chemotherapy (NACT) in an interval cytoreductive surgery (ICS) setting with the aim to propose a scoring model for predicting both complete cytoreduction and histopathologic response. **Methods:** A total of 50 consecutive patients who underwent ICS at our institution were prospectively collected between January-2020 and December-2023. PCI was assessed at exploratory surgery and at ICS. The clinical and histopathological response to NACT was determined by Δ-PCI and CRS. A cut-off value for Δ-PCI, to predict complete cytoreduction, histopathological response, and both together, was identified using a receiver operating characteristic (ROC) curve. The Kaplan–Meier test was used to define disease-free survival (DFS) based on the Δ-PCI cut-off value. **Results:** Complete cytoreduction was achieved in 82% of patients, with a median Δ-PCI score at ICS of 12 (range 7–29). The remaining 18% had a median Δ-PCI score at IDS of 8 (range 4–11). The best predictor of complete cytoreduction, histopathologic response CRS 3, and both was the Δ-PCI score, with an area under the curve (AUC) of 0.85 (0.73–0.96), 0.98 (0.94–1.00) and 0.88 (0.75–0.96), respectively; ROC curve analysis determined a Δ-PCI cut-off of 8, 17 and 15, respectively. Δ-PCI ≥ 15 as a predictor for both complete cytoreduction and histopathologic response CRS 3 with a median DFS of 26 months for Δ-PCI ≥ 15 versus 12 months for Δ-PCI < 15 (*p* = 0.02). **Conclusions:** Δ-PCI (cut-off ≥ 15) is a predictive model for complete cytoreduction, histological response CRS 3, and improved DFS.

## 1. Introduction

Ovarian cancer (OC) is the leading cause of death from gynecological malignancies, with a 5-year overall survival (OS) of approximately 40–45% [1]. With many histological variations, OC has been regarded as a highly heterogeneous disease. Epithelial ovarian cancer (EOC, 90% of OC) can be divided into two subtype groups: type I tumors that grow slowly with a distinct set of frequently mutated genes, including, KRAS, BRAF, PTEN, and CTNNB1 and include low-grade serous carcinoma (LGSC), mucinous carcinoma (MC), endometrioid carcinoma (EC), clear cell carcinoma (CCC), and type II tumors that progress rapidly with mutations in the TP53 (96%), BRCA1 and BRCA2 genes (20%) and include high-grade serous carcinoma (HGSC), and carcinosarcoma [2,3,4,5,6]. Usually, 75% of these patients are diagnosed in advanced stage with poor survival outcomes [7,8]. Unfortunately, there are no screening tests available for ovarian cancer prevention in the general population, which also results in significant healthcare costs [9]. Standard treatment for patients with advanced stages of OC consists of cytoreductive surgery, platinum/taxane-based chemotherapy and maintenance treatment. In patients where primary cytoreductive surgery (PCS) is not feasible due to extensive tumor load and/or patient comorbidities, three cycles of neoadjuvant chemotherapy (NACT) precede interval cytoreductive surgery (ICS), followed again by chemotherapy [10,11,12]. In two randomized controlled trials, ICS has been shown to be equivalent to PDS in terms of progression-free survival (PFS) and overall survival (OS) and to be associated with reduced postoperative morbidity [13,14,15,16]. ICS following NACT also provides an opportunity to assess tumor response to antineoplastic treatments [17,18]. The most important prognostic factor for PFS and OS both for PDS and ICS, is complete cytoreductive surgery (no residual disease, R0 or complete resection) leaving no gross residual disease [19]. Additionally, in the NACT setting, a prognostic role is attributed to the chemotherapy response score (CRS) that quantifies the response to NACT. CRS stratifies response to chemotherapy on histology analysis of tissue sample into complete/near complete (CRS 3), partial (CRS 2), and no/minimal (CRS 1) [20,21,22]. Despite being designed as a three-tier score, considering the similar PFS and OS, CRS 1 and CRS 2 were merged and compared to CRS 3 [22].

Based on these considerations, different scoring models have been proposed aiming to predict complete cytoreductive surgery, but no models have been proposed for histopathologic response. The peritoneal cancer index (PCI), first described by Jacquet and Sugarbaker, was introduced to quantify the extent of carcinomatosis in colorectal cancer and peritoneal mesothelioma [23,24]. Lately it has become a useful method to classify the spread of peritoneal carcinomatosis with prognostic significance in advanced ovarian cancer patients [25,26,27]. The role of PCI was largely studied for upfront surgery. On the contrary, there are limited data on the predictive power of the PCI after NACT [28,29].

The new ESGO-ESMO-ESP recommendations on OC encourage the use of chemotherapy response score (CRS) at the time of interval cytoreductive surgery (ICS), as it provides valuable prognostic information for patient outcomes. Additionally, these guidelines suggest that the peritoneal cancer index (PCI) may play a crucial role in selecting patients for upfront surgery versus ICS, helping to stratify patients who would benefit most from each approach. However, despite the importance of these indicators, there are currently no published predictive models that effectively guide clinical management in the setting of neoadjuvant chemotherapy (NACT) followed by ICS based on the assessment of tumor spread (PCI) and histopathologic response (CRS) [30].

On this basis, we analyze the role of PCI in an ICS setting, and in particular the role of PCI variation (Δ-PCI) before and after NACT with the aim of proposing an accurate scoring model for predicting both complete cytoreduction and histopathologic response.

## 2. Materials and Methods

### 2.1. Study Design and Patient Selection

We conducted an observational analysis of prospective collected data to evaluate the role of PCI and Δ-PCI calculated at initial exploratory laparoscopy (EXL) and at ICS after NACT in patients with diagnosis of advanced stage (FIGO stage IIIC or IV) OC. All consecutive patients with primary ovarian or fallopian tube tumors who underwent EXL and NACT followed by ICS at the Department of Gynaecology and Obstetrics of University of Padova between Jan-2020 and Dec-2023 were extracted from our prospective maintained data base.

The inclusion criteria were as follows: (i) PCI available at exploratory surgery and ICS; (ii) CA125 serum assay available before exploratory surgery and at ICS; and (iii) CRS analysis on omentum specimens. Exclusion criteria were as follows: (i) patients with OC who underwent upfront treatment; (ii) recurrent OC disease; (iii) patients who underwent EXL at other institutions; and (iv) lack of CA125 and CRS. Through our institution’s electronic database, for each patient, the investigators reviewed electronic hospital records and pathology reports, along with patients’ general features such as age, body mass index, menopausal status, CA125 levels, and the stage and histology of the disease.

The duration of surgery, blood loss, and complications were also retrieved. Pre-operative work-up included a full clinical assessment; serum tumor markers (CA-125); a computed tomography (CT) scan of the chest, abdomen, and pelvis; and a tissue biopsy. All patients were discussed at the multidisciplinary team meeting. At our institution, all patients with advanced OC undergo EXL. Patients are triaged to NACT if a complete resection is not achievable based on specific disease spread, which is best assessed by the combination of CT scan and EXL. After three or, occasionally, four cycles of NACT patients with a complete response (CR), partial response (PR), or stable disease (SD) to NACT based on RECIST 1.1 criteria are proposed for ICS. The latter was performed 4 weeks after the last cycle of chemotherapy aiming at complete resection. The extent of tumor involvement by PCI score was assessed during pre-operative EXL and at ICS in all patients. In addition, we calculated the difference between PCI at EXL and at ICS, defining it as Δ-PCI. Similarly, CRS in the omentum specimens derived from EXL before ICS was assessed by an experienced gynecological pathologist with Boehm’s score (MS). Despite being developed as a three-tier score, CRS 1 and CRS 2 were combined and compared as a single entity with CRS 3 as suggested in many studies due to their identical PFS and OS rates. The institutional review board of the University of Padova approved the study (IRB: 465n/AO/24).

### 2.2. Endpoints of the Study

The primary endpoint of this study was to evaluate and compare the role of PCI and Δ-PCI as a predictive model of residual disease and histopathologic response (CRS 1-2 versus CRS 3) in patients with advanced OC who have undergone NACT and ICS. Secondary endpoints included establishing a cut-off for this predictive model using a ROC curve and investigating the impact on disease-free survival (DFS) of the cut-off.

### 2.3. Statistical Analysis

The statistical analysis was performed with GraphPad Prism 10 (Version 10.1.1). Continuous variables were expressed in absolute numbers, mean ± standard deviation, or median and range. Categorical variables were expressed as absolute numbers and percentages. For categorical outcomes, chi-square (χ^2^) tests were used. The distribution of continuous outcomes was assessed with Mann–Whitney U tests and *t*-tests. The correlation between (I) PCI at IDS and Δ-PCI with residual diseases and (II) PCI at IDS and Δ-PCI with CRS was tested for significance using the Mann–Whitney U test. The strength of correlation was assessed using Spearman’s rank correlation coefficient (rS) as follows: correlation coefficient values ranging from 0.00 to 0.20 were classified as negligible correlations, from 0.21 to 0.40 as weak correlations, from 0.41 to 0.60 as moderate correlations, from 0.61 to 0.80 as strong correlations, and from 0.81 to 1.00 as very strong correlations. A peritoneal cancer index cut-off value for complete cytoreduction and histopathologic response CRS 3 was assessed with a receiver operator characteristic (ROC) curve and the Kaplan–Meier test was used for the survival analysis in term of disease-free survival, DFS, and the statistical significance of the differences between the curves was assessed using the log-rank test. For all of the statistical tests, the threshold of significance was set at 5%, and differences were considered significant if the probability of error was less than 5% (*p* < 0.05).

## 3. Results

### 3.1. Study Populations

A total of 174 patients underwent surgery for advanced OC at our institution between 2020 and 2023. Sixty-nine patients underwent neoadjuvant chemotherapy (NACT) due to unresectable widespread disease identified at EXL. Two weeks after the last NACT administration, all patients underwent a CT scan: considering their responses, patients with a CR or PR were considered eligible for ICS, while patients with PD continued with chemotherapy.

Finally, 56 patients were candidates for ICS. Among them, four patients did not undergo surgery due to significant anesthesiologic contraindications. Additionally, two patients underwent only EXL and no ICS due to diffuse carcinomatosis of the small bowel serosa not evident on CT scan, which would have required extensive resection leading to short bowel syndrome. These two patients continued chemotherapy. Thus, a total of 50 patients were included in the present study. The clinical and tumor characteristics of the study population are summarized in Table 1.

The median age at the time of surgery was 66 years (range 43–82). All women received 3–4 cycles of Paclitaxel-Carboplatin as NACT regimen. The majority of patients (45/50, 90%) had high-grade serous ovarian cancer (HGSOC). The remaining histological sub-types included ovarian clear cell (1/50, 2%), endometrioid (3/50, 6%), and carcinosarcoma (1/50, 2%). Complete cytoreduction was achieved in 41 patients (82%) while 9 patients (18%) underwent incomplete cytoreductive surgery. CRS in the omentum was scored as CRS 1-2 in 39 cases (78%) and CRS 3 in 11 cases (22%). The median intra-operative PCI score for the entire study population was 26 (range 13–36) at EXL and 15 (range 1–27) at ICS. The median Δ-PCI was 11 (range 4–29) in the study group (Table 1).

### 3.2. Correlation of PCI and Δ-PCI with Residual Disease and Histopathologic Response to Chemotherapy

#### 3.2.1. Residual Disease

**Figure 1:**
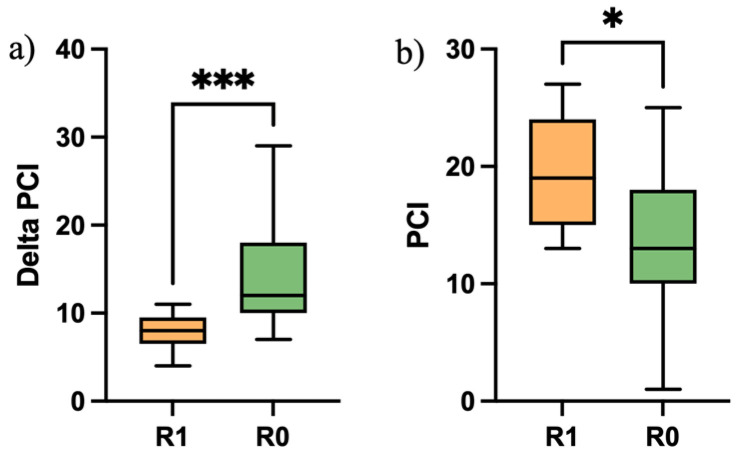
(**a**) Mann–Whitney U testing between Δ-PCI in patients with no residual disease and patients with residual disease (*p* < 0.0006, ***); (**b**) Mann–Whitney U testing between PCI score at ICS in patients with no residual disease and patients with residual disease (*p* < 0.01, *).

Patients with residual disease (incomplete cytoreduction, 18%) had a median intra-operative PCI score at pre-operative EXL of 25 (range 17–35), a median PCI at ICS of 19 (range 13–27) and a Δ-PCI of 8 (range 4–11). In patients with complete resection and no residual disease, the median intra-operative PCI score at pre-operative EXL was 27 (13–36 range), a median PCI at ICS of 13 (range 1–25) and Δ-PCI of 12 (range 7–29). The Mann–Whitney U testing showed significant differences (*p* < 0.0006) between Δ-PCI in patients with no residual disease and patients with residual disease (Figure 1a). Similarly, the Mann–Whitney U testing showed significant differences (*p* < 0.01) between PCI score at ICS in patients with no residual disease and patients with residual disease (Figure 1b).

**Figure 2:**
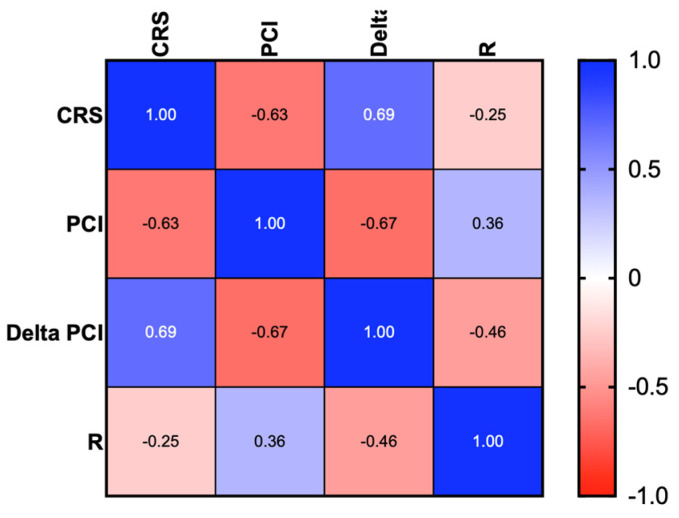
Heatmap for Spearman correlation between CRS, PCI, and Delta-PCI.

Δ-PCI showed a moderate correlation with residual disease (Spearman correlation, −0.46) with a significant difference (*p* < 0.001) while the PCI score at ICS showed weak correlation with residual disease (Spearman correlation, 0.36) by a significant difference (*p* < 0.01) (Figure 2).

#### 3.2.2. Histopathologic Chemotherapy Response (CRS)

**Figure 3:**
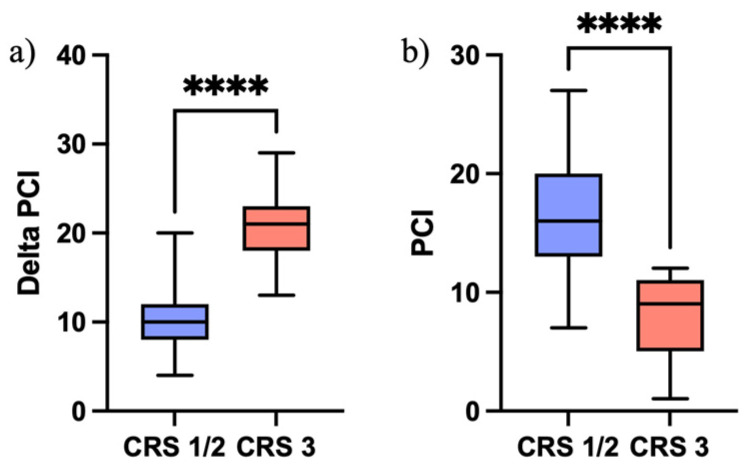
(**a**) Mann–Whitney U testing between Δ-PCI in patients with CRS 1-2 and CRS 3 (*p* < 0.0001, ****); (**b**) Mann–Whitney U testing between PCI score at ICS in patients with CRS 1-2 and CRS 3 (*p* < 0.0001, ****).

Patients with CR 1-2 had a median intra-operative PCI score at pre-operative EXL of 26 (17–36 range), a median PCI at ICS of 16 (range 7–27) and a Δ-PCI of 10 (range 4–20). Instead, patients with CRS 3 had a median intra-operative PCI score at pre-operative EXL of 29 (24–34 range), a median PCI at ICS of 9 (range 1–12) and a Δ-PCI of 21 (range 13–29). The Mann–Whitney U testing showed significant differences (*p* < 0.0001) between Δ-PCI in patients with CRS 1-2 and CRS 3 (Figure 3a). Similarly, the Mann–Whitney U testing showed significant differences (*p* < 0.0001) between PCI score at ICS in patients with CRS 1-2 and CRS 3 (Figure 3b).

These data confirmed that the chances of achieving a R0 are higher in those patients with lower tumor load (low PCI) and a better surgical and histological response to NACT. We then correlated the Δ-PCI and PCI at IDS with CRS. We showed a strong correlation between Δ-PCI with histopathologic response (Spearman correlation, r = 0.69, *p* < 0.001) (Figure 2).

### 3.3. ROC Curve Analysis for Δ-PCI as Predictor of Histopathologic Chemotherapy Response and Residual Disease

#### 3.3.1. Histopathologic Chemotherapy Response

**Figure 4:**
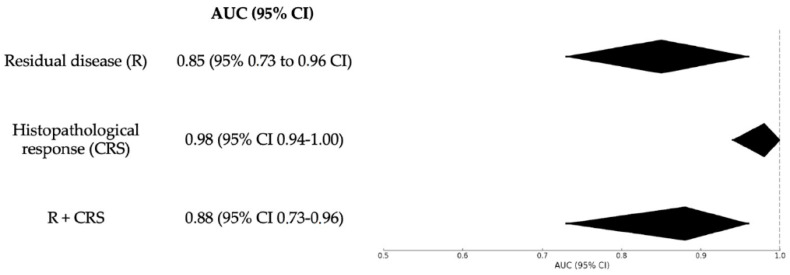
Summary forest plot of the area under the receiver operating characteristic curve (AUC) for Δ-PCI.

ROC curve analysis has been applied also to calculate the diagnostic accuracy of Δ-PCI to predict histopathologic response (CRS 1-2 and CRS 3). Δ-PCI showed an excellent accuracy with an AUC of 0.98 (95% CI 0.94–1.00) Figure 4. The best cut-off for the highest sensitivity together with the lowest false positive rate (1−specificity) of Δ-PCI was 17 (sensitivity 0.91 and specificity 0.97).

#### 3.3.2. Residual Disease

We applied ROC curve analysis to calculate the diagnostic accuracy of Δ-PCI to predict complete cytoreduction; it showed a good accuracy with an AUC of 0.85 (95% 0.73 to 0.96 CI) (Figure 4). The best cut-off for the highest sensitivity together with the lowest false positive rate (1−specificity) of Δ-PCI was 8 (sensitivity 0.77 and specificity 0.86).

#### 3.3.3. Residual Disease and Histopathologic Chemotherapy Response Combined

Finally, ROC curve analysis was applied to evaluate Δ-PCI as a predictor for complete cytoreduction and histopathologic response (CRS 3) combined. Δ-PCI showed a good accuracy with an AUC of 0.88 (95% CI 0.73–0.96) (Figure 4). The best cut-off for the highest sensitivity together with the lowest false positive rate (1−specificity) of Δ-PCI was 15 (sensitivity 0.79 and specificity 0.89).

**Figure 5:**
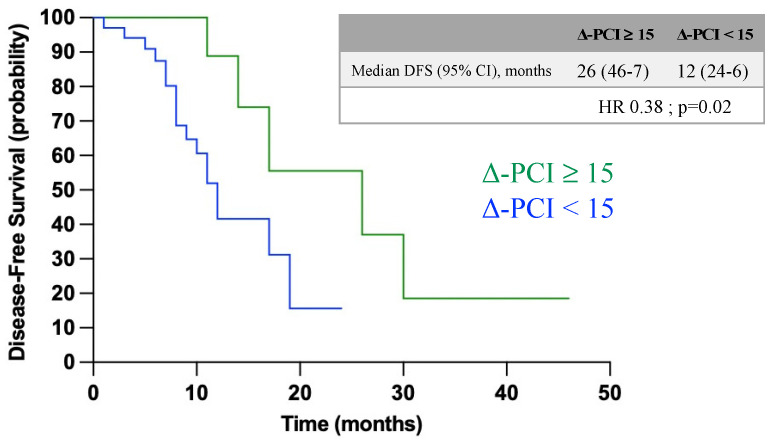
Disease-free survival (DFS) comparing patients with Δ-PCI ≥ 15 versus Δ-PCI < 15.

We then evaluated if these values were clinical meaningful by assessing disease-free survival (DFS) in patients with Δ-PCI ≥ 15 compared with those having Δ-PCI < 15 (Figure 5). The median disease-free survival for patients with Δ-PCI ≥ 15 was 26 months (95% CI 14.1–18.4), while for patients with Δ-PCI < 15 it was 12 months (95% CI = 2.8–8.3) with a hazard ratio (HR) of 0.38. The difference in DFS was a significant result (*p* = 0.020).

## 4. Discussion

Despite several studies aimed at identifying predictive markers for selecting the best candidates for upfront ovarian cancer (OC) surgery, relatively few have focused on identifying markers that can guide the selection of patients who are most suitable for interval cytoreductive surgery (ICS) following neoadjuvant chemotherapy (NACT) [31]. The evaluation of tumor response to chemotherapy has primarily relied on radiological assessments, particularly through CT scans. However, CT scans have shown significant limitations in accurately predicting the likelihood of complete cytoreduction, with wide variability in accuracy reported across the literature (refer to Table 2) [25,26,32,33,34,35,36,37,38,39,40,41,42]. In addition to the classic biomarkers (CA 125) associated with CT scans, it would be valuable in the future to identify biomarkers such as miRNA and cfDNA for the early diagnosis of ovarian cancer and its recurrence.

This lack of precision is largely due to the challenges in assessing the presence of small bowel or peritoneal deposits, which are often miliary in nature, following NACT. Such deposits can be difficult to detect, and even when they are visible, CT imaging may struggle to capture the true extent of the disease, especially when lesions have been partially reduced or when calcified residues persist post-treatment. Additionally, while CT scans can provide a general overview of tumor shrinkage, they are not always reliable in differentiating between viable tumor tissue and fibrotic or necrotic changes induced by chemotherapy.

Thus, relying solely on CT imaging may lead to inaccurate assessments of the disease burden, potentially resulting in suboptimal surgical planning. These limitations underscore the need for more accurate and reliable predictive markers—such as the Δ-PCI—that could complement or even surpass radiological evaluations, offering a more nuanced understanding of tumor response to chemotherapy and helping to better identify patients who are likely to benefit from complete cytoreduction at ICS [13,14].

In particular, two important recently published studies investigated the sensibility, specificity, and AUC in founding a CT scan–PCI cut-off for complete cytoreduction defined by CT scan. Asp et al. included a cohort of 110 PDS patients, individuating by CT scan a PCI cut-off > 21 to predict incomplete cytoreduction with a sensibility and specificity of 58% and 70% [39]. Similarly, Di Donna et al. included a cohort of 60 PDS patients, individuating by CT scan a PCI cut-off > 18 to predict incomplete cytoreduction with an AUC of 0.64; and a cohort of 31 ICS patients, individuating by CT scan a PCI cut-off > 19 to predict incomplete cytoreduction with an AUC of 0.47 [40].

Considering this low detection rate in predicting no gross residual disease after surgery using CT images, particularly in an ICS setting, some authors have introduced the use of laparoscopy in order to predict complete resection in patients with advanced OC [43,44]. This visual inspection of disease is considered a reliable predictor of complete resection, particularly in the PDS setting, allows a direct visualization of the small bowel surface and the identification of diffuse carcinosis at this level. Adding the EXL gives us the possibility to discriminate between residual disease and post-chemotherapy scars compared to the CT scan. With regards to the role of EXL in establishing the spread of disease, few studies investigated how the gross visual impression relates to the likelihood of complete resection in the NACT + ICS setting (evaluated through standardized methods like the PCI score) [26,27]. For example, Fagotti et al. [43] developed a laparoscopic scoring algorithm from 0 to 12 (predictive index, PI), including six parameters based on intra-abdominal distribution of the disease. In the final analysis, this model identified SCS for scores of 8 with a specificity of 100% (PPV 100%; NPV 70%).

Although the accuracy of the laparoscopic model is 75% at predicting surgical outcome, the percentage of unnecessary laparotomies was 33%. Adding, the modified laparoscopic PI described by Fagotti et al. [44] analyzes four variables (mesenteric retraction, bowel and stomach infiltration, and superficial liver metastases), which are associated with a high rate of suboptimal cytoreduction. External validation of this score was performed by Brun et al. [45], who reported that a laparoscopy-based score of 8 was associated with SCS, with sensitivity, specificity, and an accuracy of 46%, 89%, and 60% respectively.

On the one hand, the other score system—the PCI—considers assessment of all abdominal quadrants to identify parameter for cytoreduction. In particular, the PCI score was used in OC surgery to predict complete resection. Many studies have proposed different PCI cut-offs ranging from 10 to 24, which were linked to a higher likelihood of achieving complete cytoreduction, both in laparoscopic and laparotomic assessment (Table 2) [37,40,41,42].

Recently, Fagan et al. investigated the AUC in finding a PCI cut-off for complete cytoreduction defined by laparotomy surgery. The authors included a cohort of 100 PDS-ICS patients, individuating a PCI cut-off > 20 by laparotomy assessment to predict incomplete cytoreduction with an AUC of 0.93 [26]. Similarly, Di Donna et al. in the PDS cohort for PCI cut-off > 18, individuated to predict incomplete cytoreduction, resulted in an AUC of 0.83 and 0.73 by laparotomy and laparoscopy assessment, respectively; instead, in the cohort of 31 ICS patients for PCI cut-off > 19, individuated to predict incomplete cytoreduction, resulted in an AUC of 0.83 and 0.73 by laparotomy and laparoscopy assessment, respectively [40].

Unfortunately, most studies do not differentiate between PDS and ICS patients. Only one study introduced the concept of a Δ-PCI cut-off related to probability of achieving an R0, but this was based on a small sample size (23 patients) and did not correlate with the histopathologic chemotherapy response and with clinical outcomes [24].

### 4.1. Main Findings

In the context of interval cytoreductive surgery (ICS), pathological response to neoadjuvant chemotherapy (NACT) is recognized as a critical predictor of oncological outcomes. To enhance predictive accuracy, we developed a novel model combining an established marker of pathological response, the chemotherapy response score (CRS), with an analysis of macroscopic tumor changes before and after NACT using the peritoneal cancer index (PCI). Our findings confirmed that the PCI score at ICS serves as a significant predictor of residual disease, with a lower PCI score correlating with a higher likelihood of achieving complete cytoreduction (R0), reflected by a correlation coefficient of r = 0.36 and *p* < 0.01, and better tumor response (CRS 3).

Moreover, we found a strong correlation (r = 0.63, *p* < 0.001) between PCI at ICS and histopathological response to chemotherapy. Notably, changes in the PCI score from pre- to post-NACT (Δ-PCI) demonstrated an even stronger correlation with both residual disease (r = 0.46, *p* < 0.001) and histopathologic response (r = 0.69, *p* < 0.001), surpassing the predictive value of PCI at ICS alone.

By integrating these observations, we identified that a Δ-PCI threshold of ≥15 was a strong predictor of both complete cytoreduction (R0) and a complete pathological response to NACT (CRS 3), with an area under the curve (AUC) of 0.88. Furthermore, patients with a Δ-PCI ≥ 15 showed a significantly longer disease-free survival (DFS), averaging 26 months, compared to 12 months in those with a Δ-PCI < 15. These findings suggest that Δ-PCI could serve as a powerful predictive tool in assessing surgical and pathological outcomes following NACT, offering valuable insights for patient management and prognosis.

### 4.2. Wider Implications

Considering the latest recommendations from ESGO-ESMO-ESP, which highlight the important role of the chemotherapy response score (CRS) and the peritoneal cancer index (PCI) in selecting patients for surgery, we proposed a new novel model for use in the interval cytoreductive surgery (ICS) setting. This model aims to predict the likelihood of achieving complete cytoreduction and a histopathological response of CRS 3. Assessing the PCI, before and after NACT, might be important to evaluate early response to treatment and then to possibly triage those patients with low Δ-PCI to a further cycle of NACT and/or intraoperatively prepare them for an incomplete cytoreduction.

The integration of histopathological and macroscopic response data is particularly important because it provides a more comprehensive assessment of the tumor’s reaction to chemotherapy. This dual assessment has the potential to tailor treatment approaches on an individual basis, either escalating or de-escalating surgical and medical interventions depending on the patient’s response. By personalizing treatment strategies, the model may improve outcomes and reduce unnecessary surgical morbidity, making it an important step forward in the management of advanced ovarian cancer.

Ultimately, the combination of these factors will not only refine patient selection for surgery but also improve the overall therapeutic decision-making process, ensuring that each patient receives the most appropriate and effective treatment plan.

### 4.3. Strengths and Limitations

Our proposal is innovative because is the first study that proposed Δ-PCI integrated with both histopathological and macroscopic response in ICS setting. We included only NACT patients, making our work the most comprehensive and largest series of NACT patients dedicated to developing a predictive model for both complete cytoreduction surgery and histopathological response. We included exclusively patients referred from diagnosis to treatment to our oncological institute, excluding any sources of bias related to heterogeneous surgical choices and procedures. We presented a rigorous data collection methodology and strict inclusion criteria. This study has some obvious limitations. The first is certainly related to its retrospective design and low sample size. However, all surgical and oncological information was collected from our electronic hospital records and databases, which are compiled by clinicians at each step of patient’s treatment, certainly representing a guarantee of completeness and correctness of the data reported. The sample size is small, but still larger when compared to previous papers on this topic. Finally, our data were collected in a single oncologic center, which on one hand guarantees homogeneity and consistency, but on the other hand it may limit broad application of the findings.

## 5. Conclusions

We demonstrated for the first time in a cohort of patients who had undergone NACT followed by interval cytoreductive surgery (ICS), that the evaluation of macroscopic tumor spread before and after NACT, as measured through the peritoneal cancer index (PCI), along with its variation (Δ-PCI), is significantly associated with both histopathological response and the achievement of complete cytoreduction. By tracking these macroscopic changes in tumor burden, we were able to establish a Δ-PCI cut-off value of ≥15, which emerged as a strong predictor of complete cytoreduction (R0), a chemotherapy response score (CRS) of 3, and improved disease-free survival (DFS).

Our findings are highly relevant as they suggest that Δ-PCI could serve as a simple yet powerful clinical tool to guide patient management in the NACT and ICS settings. A Δ-PCI cut-off of ≥15 offers the ability to predict not only surgical outcomes but also long-term oncological benefits such as improve DFS. If these results are confirmed through further prospective analyses, the Δ-PCI could become a valuable metric for clinicians to tailor treatment strategies more effectively, optimizing both surgical and medical interventions based on individual patient response to chemotherapy.

The potential of this tool lies in its accessibility in clinical practice. By incorporating Δ-PCI into routine assessments, clinicians could make more informed decisions regarding the necessity of additional NACT cycles, the likelihood of achieving complete cytoreduction, and the overall prognosis. This would allow for a more personalized approach to treatment, potentially reducing the risk of unnecessary surgical morbidity in patients unlikely to achieve complete cytoreduction while enhancing the chances of achieving optimal outcomes in those who are responsive to chemotherapy.

## Figures and Tables

**Table 1:** Baseline characteristics.

	Complete Cytoreduction	Incomplete Cytoreduction	Total	*p* Value
	*n* = 41 (82%)	*n* = 9 (18%)	*n* = 50	
Mean Age (years)	66 (43–82)	67 (57–75)	66 (43–82)	
Mean CA 125 (U/mL)				0.06
Pre NACT	1767 (242–1380)	1254 (340–4414)	1673 (242–4414)	
Post NACT	348 (9–883)	440 (28–2164)	365 (9–2164)	
FIGO stage				0.9
III	32 (78%)	7 (55%)	39 (78%)	
IV	9 (22%)	2 (22%)	11 (22%)	
Surgical complications				0.04
No complications	33 (81%)	6 (67%)	39% (78%)	
Clavien–Dindo < 3	7 (17%)	2 (22%)	9 (18%)	
Clavien–Dindo ≥ 3	1 (2%)	1 (11%)	2 (4%)	
CRS				0.08
CRS 1/2	30 (73%)	9 (100%)	39 (78%)	
CRS 3	11 (27%)	-	11 (22%)	
Median PCI (Range)				0.01
Before NACT	27 (13–36)	25 (17–35)	26 (13–36)	
ICS	13 (1–25)	19 (13–27)	15 (1–27)	
Delta PCI	12 (7–29)	8 (4–11)	11 (4–29)	
BRCA status				0.06
Mut	6	4	10	
WT	35	5	40	

NACT: neoadjuvant chemotherapy; CRS: chemotherapy response score; PCI: peritoneal cancer index.

**Table 2:** Literature review of studies comparing CT scan and surgical assessment in predicting R0 probability, including PCI and ΔPCI cut-off analysis.

Study	Methods	Patients	Sensitivity%	Specificity%	AUC	Cut-Off
			(CI 95%)	(CI 95%)		
Nelson et al., 1993 [32]	CT scan	51 PCS	92.3	79.3	-	-
Meyer et al., 1995 [33]	CT scan	28 PCS	58	100	0.94	-
Bristow et al., 2002 [34]	CT scan	81 PCS	100	85	-	-
Tentes et al., 2003 [35]	CT scan	60 PCS	-	-	-	PCI > 10
Dowdy et al., 2004 [36]	CT scan	87 PCS	64	81	-	-
Llueca et al., 2018 [37]	Laparoscopy	80 PDS	38	88	0.73	
	Laparotomy		73	81	0.83	
	CT scan		27	91	0.64	
Elzarkaa et al., 2018 [41]	Laparotomy	96 PCS	80.6	45	0.64	PCI > 20
Avesani et al., 2020 [38]	CT scan	297 PCS/ICS	-	-	0.64	
Jónsdóttir et al., 2021 [42]	Laparotomy	167 PDS	100	73.6	0.94	PCI > 13
Asp M et al., 2022 [39]	CT scan	110 ICS	58.5	70.3	-	PCI > 16
Rawert et al., 2022 [25]	Laparoscopy	23 ICS	88	67	0.82	PCI > 24
Di Donna et al., 2023 [40]	Laparoscopy	60 PCS	-	-	0.83	PCI > 21
	Laparotomy		-	-	0.73	
	CT scan		-	-	0.64	
	Laparoscopy	31 ICS	-	-	0.76	PCI > 17, Δ-PCI > 8.5
	Laparotomy		-	-	0.87	
	CT scan		-	-	0.47	
Fagan et al., 2023 [26]	Laparotomy	100 PCS/ICS	-	-	0.93	PCI > 18

CT: computed tomography; PCI: peritoneal cancer index; PCS: primary cytoreductive surgery; ICS: interval cytoreductive surgery.

## Data Availability

The data presented in this study are available upon request from the authors.
